# *In vitro* activities of antifungals alone and in combination with tigecycline against *Candida albicans* biofilms

**DOI:** 10.7717/peerj.5263

**Published:** 2018-07-25

**Authors:** Mayram Hacioglu, Ayse Seher Birteksoz Tan, Sibel Dosler, Nese Inan, Gulten Otuk

**Affiliations:** 1 Department of Pharmaceutical Microbiology, Istanbul University, Istanbul, Turkey; 2 Department of Microbiology, Faculty of Medicine, Istanbul Bilim University, Istanbul, Turkey

**Keywords:** Biofilm, Antifungal, Tigecycline, XTT, *Candida albicans*

## Abstract

**Background:**

*Candida* may form biofilms, which are thought to underlie the most recalcitrant infections.

**Methods:**

In this study, activities of antifungal agents alone and in combination with tigecycline against planktonic cells and mature and developing biofilms of *Candida albicans* isolates were evaluated.

**Results:**

Amphotericin B and echinocandins were found to be the most effective agents against mature biofilms, whereas the least effective agent was fluconazole. Furthermore, the most effective anti-fungal monotherapies against biofilm formation were amphotericin B and anidulafungin, and the least effective monotherapy was itraconazole. The combination of tigecycline and amphotericin B yielded synergistic effects, whereas combinations containing itraconazole yielded antagonist effects against planktonic cells. The combination of tigecycline and caspofungin exhibited maximum efficacy against mature biofilms, whereas combinations containing itraconazole exhibited minimal effects. Combinations of tigecycline with amphotericin B or anidulafungin were highly effective against *C. albicans* biofilm formation.

**Discussion:**

In summary, tigecycline was highly active against *C. albicans* particularly when combined with amphotericin B and echinocandins.

## Introduction

In humans, especially in immunocompromised individuals, *Candida albicans* is both a commensal organism and one of the most important opportunistic fungal pathogen, (Kim & Sudbery, 2011). Invasive candidiasis is considered a life-threatening infection associated with high morbidity and mortality (Pappas et al., 2016). Many invasive *C. albicans* infections can be attributed to the ability of this organism to form biofilms on host tissues and medical devices implanted in a patient’s body. These biofilms are characterised by high levels of resistance to both conventional antifungal drug therapies and host immune defences, and therefore represent a major challenge to the therapeutic use of catheters and medical devices (Douglas, 2003; Nett & Andes, 2006). In affected patients, *Candida* infections are difficult to resolve without removing and/or replacing devices through undesirable and/or high-risk procedures (Ramage, Martínez & López-Ribot, 2006).

Lipid formulations of amphotericin B, two triazole agents (voriconazole and posaconazole) and echinocandins (anidulafungin, caspofungin and micafungin) are the major antifungal agents used to treat and prevent *Candida* infections. However, susceptibility studies indicate that *C. albicans* biofilms may be up to 1,000 times more resistant than planktonic cells to these agents (Shuford et al., 2007; Tobudic et al., 2010; Tobudic et al., 2012; Sardi et al., 2013). New strategies, therapies and synergistic drug combinations are therefore needed to combat biofilm-related infections.

The antibiofilm activities of non-antifungal drugs are currently under investigation. Notably, synergistic antimicrobial activities against *C. albicans* planktonic cells and biofilms were observed when conventional antifungal agents were combined with non-antifungals, including antibacterials, quorum-sensing molecules, calcineurin inhibitors, Hsp90 inhibitors and calcium homeostasis regulators (El-Azizi, 2007; Gamarra et al., 2010; Shinde et al., 2012; Shinde et al., 2013; Xia et al., 2017). In addition, previous studies demonstrated that at high concentrations, some tetracycline derivatives exhibited antimicrobial activities against *Candida* species. Tetracyclines inhibit mRNA translation in bacteria by binding to the 30S ribosomal unit and can also disturb protein synthesis in the mitochondria of eukaryotic cells, as the mitochondrial ribosome is related structurally and functionally to the bacterial ribosome (Chopra & Roberts, 2001). Therefore, some tetracycline derivatives are slightly active against *Candida* spp. (Hooper, Ashcraft & Pankey, 2018; Liu et al., 2014). Ku, Palanisamy & Lee (2010) showed that a combination of high concentrations of tigecycline with fluconazole, amphotericin B and caspofungin enhanced the activities of antifungal agents at different concentrations. Other reports also showed that high concentration of doxycycline may be useful against *C. albicans* and non-*albicans Candida* species when combined with traditional antifungals (El-Azizi, 2007; Miceli, Bernardo & Lee, 2009). However, in the literature no available data describe the effects of combinations of tigecycline and anidulafungin or itraconazole against *Candida* biofilms. In this study, we sought to determine the *in vitro* effects of traditional antifungals, both alone and in combination with high-concentration tigecycline, against planktonic cells and developing and mature biofilms of clinical *C. albicans* strains.

## Materials and Methods

### Organisms

*Candida albicans* isolates were obtained from blood submitted to the Clinical Microbiology Laboratories of Group Florence Nightingale Hospitals in Turkey, single sample per person. Isolates were identified with Vitek 2 (BioMerieux, Craponne, France) and verified with API 20 C AUX (BioMerieux, France). The clinically derived biofilm producing wild-type strain *C. albicans* SC5314 and biofilm producing clinical isolates (*n* = 15) were studied.

### Antimicrobial agents

Amphotericin B, itraconazole, caspofungin and tigecycline were obtained from Bristol-Myers Squibb (New York, USA), Sigma Aldrich (St. Louis, MO, USA), Merck Sharp Dohme (Kenilworth, NJ, USA) and Wyeth Pharmaceuticals (Madison, NJ, USA), respectively, while fluconazole and anidulafungin were acquired from Pfizer (New York, USA). Stock solutions from dry powders were prepared according to the manufacturers’ recommendation and stored frozen at −80 °C for up to six months. Final concentrations of antimicrobials were prepared in Roswell Park Memorial Institute (RPMI, Sigma-Aldrich, St. Louis, MO, USA) medium supplemented with L-glutamine and buffered with morpholinepropanesulfonic acid (MOPS; Sigma-Aldrich, St. Louis, MO, USA), prior to use.

### Medium

Sabouraud dextrose broth (SDB, Difco, Sparks, MD, USA) supplemented with 8% glucose was used to investigate biofilm production. Yeast extract peptone dextrose (YPD, Sigma-Aldrich, St. Louis, MO, USA) agar and YPD broth were used for biofilms formation. Sabouraud dextrose agar (SDA, Difco, Sparks, MD, USA) and RPMI 1640 medium, were used to determine the minimum inhibitory concentrations (MIC) and sessile minimal inhibitory concentrations (SMIC) and at biofilm formation assays.

### MIC determinations

The MICs of antimicrobial agents against planktonic cells were determined by Clinical and Laboratory Standards Institute (CLSI) M27-A3 broth microdilution method (CLSI, 2008). *C. albicans* ATCC 90028 and ATCC 10231 were also used as quality control. Each isolate was placed on SDA and incubated at 37 °C for 24 h. The yeast inoculum was adjusted to a concentration 1 × 10^3^–5 × 10^3^ cells ml^−1^ in RPMI 1,640 medium. Dilutions of caspofungin (0.001–1 µg ml^−1^), tigecycline (10–5,120 µg ml^−1^) and other antifungals (0.06–32 µg ml^−1^) were prepared and tested. The microtitre plates were incubated at 35 °C for 24–48 h.

The lowest concentration inhibiting any discernible growth at 48 h was used as the MIC for amphotericin B whereas the lowest concentration associated with 50% reduction in growth turbidity compared with the control well at 24 h was used as the MIC for anidulafungin and caspofungin and at 48 h was used as the MIC for fluconazole, itraconazole and tigecycline. Experiments were performed in duplicates.

MIC was determined for each antifungal agent and used to classify the susceptibility of the isolates for fluconazole ≤8 µg ml^−1^ susceptible, MIC between 16 and 32 µg ml^−1^ susceptible dose-dependent and MIC ≥64 µg ml^−1^ resistant, for anidulafungin and caspofungin it was evaluated MIC ≤2 µg ml^−1^ susceptible and MIC >2 µg ml^−1^ resistant and for itraconazole ≤0.125 µg ml^−1^ susceptible, MIC between 0.25 and 0.5 µg ml^−1^ susceptible dose-dependent and MIC ≥1 µg ml^−1^ resistant. Due to the lack of defined breakpoints for amfotericin B it was compared with the results of other investigations (CLSI, 2008).

### Antifungal and tigecycline combinations against planktonic cells

Interactions between antifungals and tigecycline were determined by the microbroth checkerboard technique (Pillai, Moellering Jr & Eliopoulos, 2005). Dilutions of individual drugs and their different combinations were prepared in checkerboard format. After incubation at 35 °C for 48 h, the fractional inhibitory concentration index (FICI) was determined as the combined concentration of antimicrobials divided by the single concentration. The combination value was derived from the highest dilution of the antimicrobial combination that permitted no visible growth. With this method, a FICI of ≤0.5 was considered synergistic, of >0.5–4 was considered indifferent, and of >4.0 was considered antagonistic (Odds, 2003). Experiments were performed in duplicates.

### Biofilm formation

Biofilms were formed in the wells of microtiter plates as previously described by Ramage et al. (2001). Overnight cultures of isolates from a 24 h growth of YPD agar were inoculated in YPD broth, in an orbital shaker at 30 °C overnight. Cultures were centrifuged (about 3,000 rpm, 5–10 min) and washed twice with sterile physiological buffered saline (PBS) and resuspended in RPMI 1,640 to a cellular density equivalent to 1 × 10^6^ cells ml^−1^. Biofilms were formed by pipetting 200 µl of the standardized cell suspension, into selected wells of polystyrene flat-bottomed 96-well tissue culture microtitre plates (Greiner Bio-One, Kremsmuenster, Austria) and incubated for 48 h at 37 °C. After incubation, the waste medium was aspirated gently, and non-adherent cells were removed by washing the biofilms three times with PBS.

### Biofilm formation quantification

Biofilm formation of fifteen clinical *C. albicans* strains were quantified by crystal violet assay described by others (Djordjevic, Wiedmann & McLandsborough, 2002). Briefly, after biofilm formation, each well was washed twice with 200 µl of PBS and air dried for 45 min. Then, each washed well was stained with 110 µl of 0.4% aqueous crystal violet solution for 45 min. Afterwards, each well was washed four times with 350 µl of sterile distilled water and immediately distained with 200 µl of 95% ethanol. After 45 min of distaining, 100 µl of distaining solution was transferred to a new well and the amount of the crystal violet stain in the distaining solution was measured with a microtiter plate reader (BioRad Novapath) at 595 nm. The absorbance values of the negative controls (containing no cells) were subtracted from the values of the test wells to minimize background interference. Each strain was tested six times and biofilm production quantities were reported as the arithmetic mean of absorbance values of the six replicate tests.

### SMIC determinations

Activities of antimicrobial agents on mature *C. albicans* biofilms, were studied using the standardized static microtitre plate model and measured by 2,3-bis (2-methoxy-4-nitro-5-sulfophenyl)-5-[8phenylamino) carbonyl]-2H-tetrazolium hydroxide (XTT; Sigma-Aldrich, St. Louis, MO, USA) reduction assay which is the most commonly test used to estimate viable biofilm growth (Pierce et al., 2010). Doubling concentrations of antimicrobials were added to the pre-formed 48 h mature biofilms, as described above. Drug-free biofilm wells containing only RPMI 1,640 were used as controls. Biofilms were incubated at 37 °C for 48 h. After incubation, the medium was aspirated and washed with PBS, three times. XTT was prepared as previously published and added to each well. Microtitre plates were incubated in the dark for 3 h at 37 °C. Biofilm growth was measured spectrophotometrically at optical density 450 nm, on microplate reader (BioRad Novapath). SMICs were determined as the minimum antifungal drug concentration that caused 50% reduction of biofilm compared to drug-free untreated biofilm controls. Each experiment was performed in four wells and was repeated two times.

### Antifungal and tigecycline combinations against mature biofilm

*Candida albicans* mature biofilms were formed as described above. Interactions between antifungals and tigecycline (SMICs for antifungals and 512 µg ml^−1^ for tigecycline) on mature biofilms were determined by the microbroth checkerboard technique (Pillai, Moellering Jr & Eliopoulos, 2005). Dilutions of individual drugs and their different combinations were prepared in a checkerboard format. After incubation at 37 °C for 48 h, the medium was aspirated and washed with PBS, then measured with XTT reduction assay as described above. Experiments were performed in duplicates.

### Inhibition of biofilm formation

*Candida albicans* strains (1 × 10^6^ cells ml^−1^) were added to each well of 96-well tissue culture microtitre plate with MIC, 10^−1^× MIC and 10^−2^× MIC of antifungals and tigecycline. The positive controls without antimicrobial agent and negative controls without cells were also added. The plates were incubated for 24 and 48 h at 37 °C. After incubation, the wells were washed twice with PBS and measured in PBS at 450 nm on microplate reader (Bio-Rad Novapath). Inhibition of biofilm formation was determined with comparing results with positive controls (Dosler & Karaaslan, 2014). Each experiment was performed in four wells and was repeated two times.

### Antifungal and tigecycline combinations on inhibition of biofilm formation

*Candida albicans* strains (1 × 10^6^ cells ml^−1^) were added to each well of 96-well tissue culture microtitre plate with MIC and sub-MICs of antifungals and tigecycline, as described above. Interactions between antifungals and tigecycline on pre-formed biofilms were determined by the microbroth checkerboard technique (Pillai, Moellering Jr & Eliopoulos, 2005). The positive controls without antimicrobial agent and negative controls without cells were also added. The plates were incubated for 48 h at 37 °C. After incubation, the wells were washed twice with PBS and measured in PBS at 450 nm on microplate reader (Bio-Rad Novapath). Inhibition of biofilm formation was determined with comparing results with positive controls (Dosler & Karaaslan, 2014). Experiments were performed in duplicates.

### Statistical analysis

All experiments were performed in duplicate. All data were expressed as mean values with corresponding standard deviations. *t* test was used to compare the differences between control and treatment and a *p*-value of <0.05 was considered statistically significant. Statistical analyses were performed with GraphPad Prism 5.0 (GraphPad Software Inc., San Diego, CA, USA).

## Results

### Biofilm formation quantification by crystal violet staining

**Figure 1:**
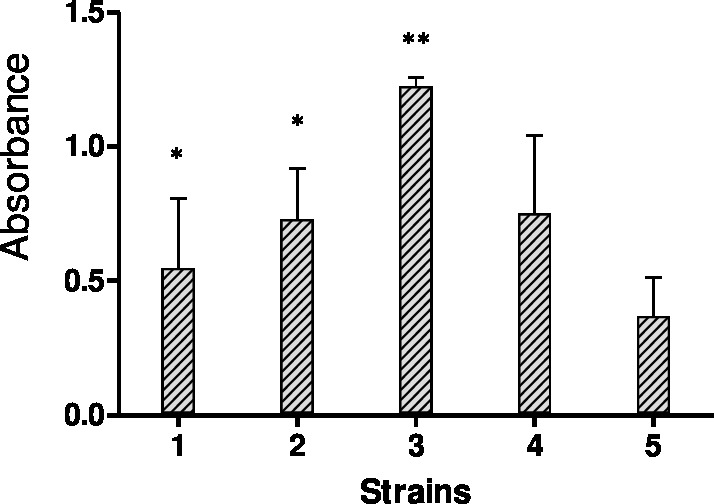
Results of crystal violet staining. Each graph represents data of the mean of individual biofilms (OD595 nm). Each experiment was performed six times and biofilm production quantities are reported as the arithmetic mean of absorbance values.

The biofilms were stained using the crystal violet staining method to quantify bulk biofilm production. Since a total of four out of fifteen strains showed off-scale absorbance values (OD595), further dilutions were made to obtain the actual absorbance value. Figure 1 shows biofilm quantification by crystal violet staining for five Candida species, *C. albicans* SC5314 and four strong biofilm produced clinical isolates.

### MICs and SMICs of antimicrobial agents

The MICs and SMICs of the five antifungals and tigecycline against *C. albicans* strains are shown in Table 1. Planktonic isolates were susceptible to all antifungal agents studied. The MICs of amphotericin B, fluconazole, itraconazole, anidulafungin and caspofungin were between 0.25–1, 0.25–0.5, 0.125–0.25, 0.06–0.25 and 0.0005–0.125 µg ml^−1^, respectively, none of the isolates were found to be resistant to antifungals.

**Table 1:** MICs (µg ml^−1^) and SMICs (µg ml^−1^) of antimicrobial agents against *Candida albicans* strains.

	AMB		FLC		ITC		ANI		CAS	
	MIC	SMIC	MIC	SMIC	MIC	SMIC	MIC	SMIC	MIC	SMIC
*C.albicans* 1	1	1	0.5	32	0.25	0.5	0.06	64	0.06	0.5
*C.albicans* 2	1	4	0.25	32	0.125	2	0.25	0.25	0.0005	0.25
*C.albicans* 3	1	16	0.5	>1024	0.25	2	0.06	32	0.03	0.5
*C.albicans* 4	0.25	8	0.25	1024	0.125	8	0.125	0.25	0.001	0.125
*C.albicans* SC5314	0.5	8	0.5	8	0.25	8	0.06	2	0.125	0.25

**Notes.**

AMB Amphotericin B FLC Fluconazole ITC Itraconazole ANI Anidulafungin CAS Caspofungin

Biofilms were resistant to high concentrations of the most of the drugs and SMICs of amphotericin B, fluconazole, itraconazole, anidulafungin and caspofungin were found between 1–16, 8–>1,024, 0.5–8, 0.25–64 and 0.125–0.5 µg ml^−1^, respectively. It was found that amphotericin B and echinocandins were the most effective agents against mature biofilms, whereas fluconazole was the least. Tigecycline, when used alone, had no antifungal activity at the concentrations employed (up to 2,560 µg ml^−1^ at planktonic cells and up to 10,240 µg ml^−1^ at biofilm cells) against any of the *Candida* strains tested.

### Antifungal and tigecycline combinations against planktonic cells

The combination of antifungals and tigecycline were tested against five *C. albicans* strains by checkerboard method. As pointed in Table 2, four amphotericin B and tigecycline combinations (FICI values 0.375) and one caspofungin and tigecycline combinations (FICI value 0.18) showed synergistic effect. Besides, this some of the itraconazole, fluconazole and caspofungin combinations showed antagonist effect against *C. albicans* strains.

**Table 2:** *In vitro* activities of antifungals and tigecycline combinations against planktonic cells. The FICI was determined as the combined concentration of antimicrobials divided by the single concentration. The combination value was derived from the highest dilution of the antimicrobial combination that permitted no visible growth. FICI of ≤0.5 was considered synergistic, of  >0.5–4 was considered additive indifferent, and of  >4.0 was considered antagonistic.

	FIC index				
	AMB	FLC	ITC	ANI	CAS
*C.albicans* 1	0.375	5	1.5	2.5	5
*C.albicans* 2	0.375	2	1	0.56	1.5
*C.albicans* 3	0.325	2	2.5	2	3
*C.albicans* 4	0.56	1	5	1	1.5
*C.albicans* SC5314	0.375	2	5	1	0.18

**Notes.**

AMB Amphotericin B FLC Fluconazole ITC Itraconazole ANI Anidulafungin CAS Caspofungin

### Antifungal and tigecycline combinations on mature biofilms

The *in vitro* activities of combinations against mature biofilms were investigated with checkerboard assays and results are shown at Table 3. It was found that, the SMIC values of amphotericin B and echinocandins decreased up to eightfold with presence of tigecycline. Nevertheless SMICs of azoles increased by up to eightfold, in the presence of tigecycline.

**Table 3:** *In vitro* activities of antifungals and tigecycline combinations against mature biofilms.

	Interactions on mature biofilm in SMIC									
	AMB		FLC		ITC		ANI		CAS	
	D/I	µg ml^−1^	D/I	µg ml^−1^	D/I	µg ml^−1^	D/I	µg ml^−1^	D/I	µg ml^−1^
*C.albicans* 1	2×	0.5	2×	16	(+)2×	1	1×	64	2×	0.25
*C.albicans* 2	2×	2	(+)8×	256	(+)8×	16	(+)4×	1	4×	0.06
*C.albicans* 3	2×	8	1×	>1024	(+)2×	4	1×	32	8×	0.06
*C.albicans* 4	1×	8	1×	1024	1×	8	2×	0.125	1×	0.125
*C.albicans* SC5314	2×	4	1×	8	1×	8	1×	2	1×	0.25

**Notes.**

D/I: Decrease or Increase 1×, no significant decrease; 2–8×, 2–8 fold decrease in SMIC. (+)2–8 fold increase in SMIC.

AMB Amphotericin B FLC Fluconazole ITC Itraconazole ANI Anidulafungin CAS Caspofungin

### Inhibition of biofilm formation

**Figure 2:**
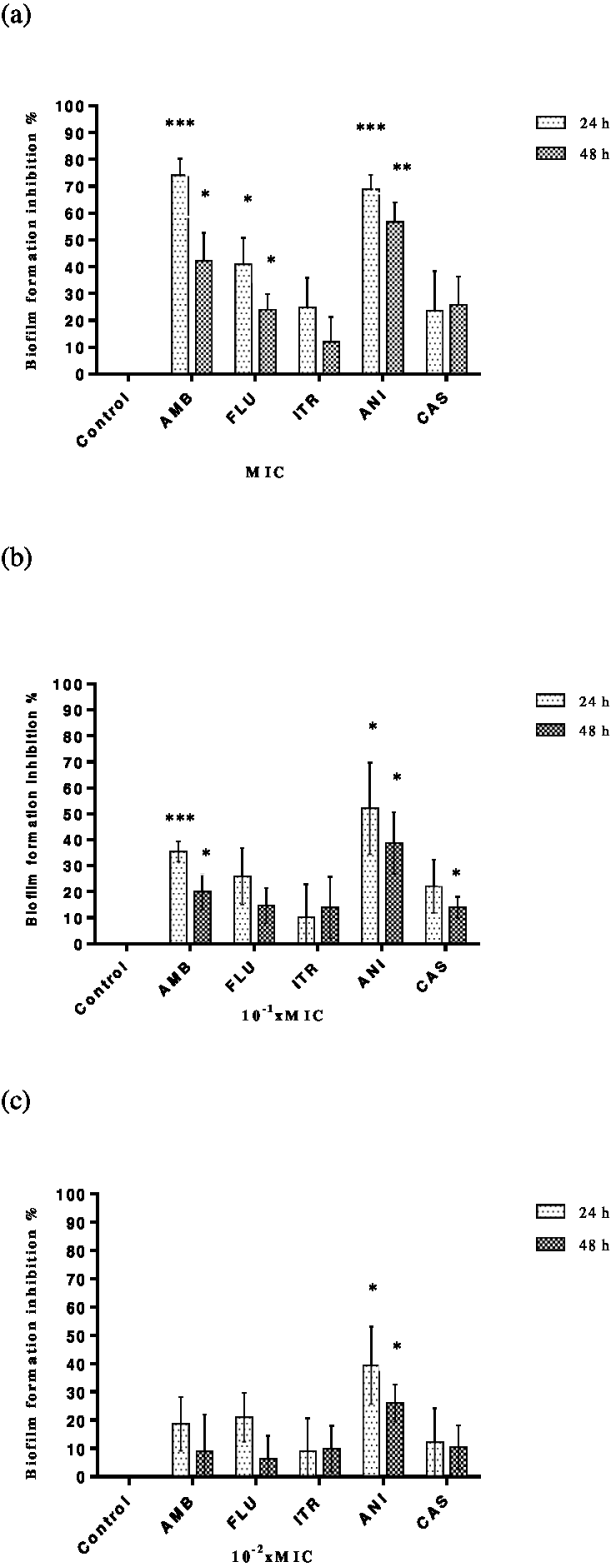
Inhibition of *C. albicans* biofilm formation by antifungals. Control bars indicate cells without any antifungal, accepted as 0% inhibition. Plates contained (A) MIC, (B) 10^−1^ × MIC and (C) 10^−2^ × MIC of antifungal and inoculums of 1 ×10^6^ cells ml^−1^ *C. albicans* were incubated for 24 or 48 h at 37 °C. Each experiment was performed in four wells and was repeated two times. Each bar represents the mean value of these four wells. Inhibition of biofilm formation at (A) MIC, (B) 10^−1^ × MIC and (C) 10^−2^ × MIC

MICs and sub-MICs of antifungals alone were tested against *C. albicans* strains to assess the biofilm development. Each result represents the mean of the five strains of *C. albicans* tested for each antimicrobial. Although inhibition of adhesion rates depended on time and concentration, it was found that the range of percentage inhibition rates for amphotericin B, fluconazole, itraconazole, anidulafungin and caspofungin were between 8.9–74.06%, 6.24–40.87%, 9–24.8%, 26.07–68.89% and 10.42–25.75%, respectively. As expected, the highest inhibition of adhesion was determined at MIC and the lowest rates at 10^−2^× MIC. Amphotericin was found the most effective agent at MIC and anidulafungin at sub-MICs (Figs. 2A–2C). Tigecycline was showed no significant reduction of inhibition of biofilm formation against *C. albicans* strains both MIC and sub-MICs.

### Antifungal and tigecycline combinations on inhibition of biofilm formation

The *in vitro* activities of antifungals with tigecycline combination were also tested against *C. albicans* biofilm formation. It was found that with presence of tigecycline, amphotericin B and anidulafungins’ MICs were reduced by two-to eightfold (Table 4).

**Table 4:** *In vitro* activities of antifungals and tigecycline combinations against biofilm formation 2–8×, 2–8 fold decrease in SMIC. (+)2–8 fold increase in SMIC.

	Interactions on biofilm formation in MIC									
	AMB		FLC		ITC		ANI		CAS	
	D/I	µg ml^−1^	D/I	µg ml^−1^	D/I	µg ml^−1^	D/I	µg ml^−1^	D/I	µg ml^−1^
*C.albicans* 1	4×	0.25	(+)2×	1	1×	0.25	1×	0.06	1×	0.06
*C.albicans* 2	8×	0.125	1×	0.25	1×	0.125	2×	0.125	(+)2×	0.001
*C.albicans* 3	2×	0.5	1×	0.5	1×	0.25	2×	0.03	1×	0.03
*C.albicans* 4	2×	0.125	1×	0.25	1×	0.125	8×	0.015	1×	0.001
*C.albicans* SC5314	2×	0.25	1×	0.5	1×	0.25	4×	0.015	1×	0.125

**Notes.**

D/I: Decrease or Increase 1×, no significant decrease; 2–8×, 2–8 fold decrease in MIC. (+)2 fold increase in MIC

AMB Amphotericin B FLC Fluconazole ITC Itraconazole ANI Anidulafungin CAS Caspofungin

All antifungal and tigecycline combination assays results were strain dependent.

## Discussion

*Candida* species are the fourth-most common cause of hospital-acquired bloodstream infections in most of the developed world (Pappas et al., 2016). The formation of biofilms on living and non-living surfaces has been associated with *C. albicans* pathogenesis, as these forms are better protected than free-living cells from immune defense and antimicrobial agents (Nett & Andes, 2006). One of the most important features of *Candida* biofilms is the high level of resistance to various antifungal agents currently in clinical use, especially azoles and polyenes (Ramage et al., 2012). This study also confirmed this strong resistance to antifungal drugs, especially azoles.

Fungal biofilms have been reported to be up to 1,000 times more resistant to antifungal agents than their planktonic cell counterparts, largely due to the physiological state within the biofilm, which includes an increased cell density, quorum sensing, over expression of drug targets, efflux pumps and extracellular matrix, persistent cells and stress responses (Ramage et al., 2012; Taff et al., 2013). Several recent *in vitro* studies of *C. albicans* biofilms have demonstrated efficient antifungal activities of amphotericin B and echinocandins, including caspofungin and anidulafungin, relative to other traditional antifungal agents (Kuhn et al., 2002; Katragkou et al., 2008; Tobudic et al., 2010; Marcos-Zambrano et al., 2016). As predicted by earlier works, this study also highlighted the efficacy of amphotericin B and echinocandin monotherapies against all but one strain, and observed a SMIC of 64 µg ml^−1^ for anidulafungin. For this strain gene mutations can be evaluated in future investigations, to find out the reason of this difference. Azoles alone exerted only modest activity against *C. albicans* biofilms.

Through antifungal susceptibility studies of clinical isolates of *C. albicans*, Hawser & Douglas (1994) were the first to demonstrate that biofilm formation affects susceptibility to antifungal agents, with corresponding high SMICs. Accordingly, when the antibiofilm activities of antifungals were considered, it was observed that SMIC/MIC ratios of amphotericin B, fluconazole, itraconazole, anidulafungin and caspofungin ranged from 1 to 32, 16 to >4096, 2 to 64, 1 to 1024 and 2 to 500, respectively. Consistent with these findings, Fiori et al. (2011) tested the activities of antifungal agents against clinical isolates of *C. albicans* and obtained high SMIC_90_ values for fluconazole, voriconazole and amphotericin B, but very low values for echinocandins. Similarly, Shuford et al. (2007) also reported higher SMIC values of amphotericin B, voriconazole and caspofungin against sessile *C. albicans*.

Novel approaches to biofilm control might take one of three main forms: effective reduction of planktonic cells before biofilm formation, inhibition of cell adhesion and biofilm formation or removal of established biofilm (Dosler & Karaaslan, 2014). The presence of biofilms on medical devices, particularly catheters, makes treatment difficult. Although current recommendations suggest the removal of these devices, clinicians frequently consider this option difficult because of the patients’ underlying conditions (Ramage, Martínez & López-Ribot, 2006). A novel field of research has accordingly focused on preventing biofilm development and adherence. In this study, it was investigated the *in vitro* activities of five conventional antifungals and tigecycline against the formation and adhesion of biofilms of various *C. albicans* strains, using the MIC or sub-MIC values of antimicrobials. Notably, it was observed that antimicrobials inhibited biofilm formation in a concentration and time dependent manner. Although all antifungal monotherapies inhibited biofilm formation by up to 75% at MIC values and up to 52% at sub-MIC values, amphotericin B and anidulafungin were the most active agents, whereas itraconazole was the least active. Effects of antifungals were found similar on mature biofilm.

The eradication of mature biofilms is extremely difficult, as predicted by this work and demonstrated by other investigators. Therefore, multiple attempts, including antimicrobial lock therapy (ALT), have been made to eradicate biofilm formation and improve the management of catheter-related infections. ALT is based on the installation of highly concentrated antimicrobial agents into the lumens of catheter. These agents are intended to sterilise the catheter, thus preventing or treating related bloodstream infections and avoiding removal procedures (Walraven & Lee, 2013). Various antimicrobials, including tetracyclines and derivatives (e.g., tigecycline), have been evaluated as potential antimicrobial lock solutions against catheter-related bloodstream infections and biofilms (Aslam et al., 2007; Bookstaver et al., 2009; Miceli, Bernardo & Lee, 2009; Ku, Palanisamy & Lee, 2010). Using the ALT principle, it was also challenged planktonic cells and mature and developing biofilms with combinations of antifungals and high-dose tigecycline according to antimicrobial MIC or sub-MIC values.

This study demonstrated that antifungal agents were more active against *C. albicans* when administered in combination with high concentrations of tigecycline, rather than monotherapies. Furthermore, synergistic effects against planktonic cells were observed with almost all combinations involving the antifungal drug amphotericin B. Moreover, a combination of amphotericin B and tigecycline exerted significant antimicrobial effects against both mature and developing biofilms. Tigecycline also enhanced the antifungal activities of echinocandins against mature and developing biofilms of *C. albicans*. Tigecycline is a derivative of tetracycline, a member of a class of antimicrobial drugs with a broad spectrum of antibiotic activity. Some tetracycline derivatives were previously reported to exhibit slight efficacy against *Candida* spp. (Hooper, Ashcraft & Pankey, 2018; Liu et al., 2014). For example, Miceli, Bernardo & Lee (2009) observed that doxycycline, when administered as a monotherapy at concentrations up to 2,048 µg ml^−1^, could reduce biofilm metabolic activity by up to 89.1%. Administration of this agent in combination with amphotericin B (512 µg ml^−1^) enhanced the antifungal activity of the latter at low concentrations. El-Azizi (2007) demonstrated that a combination of doxycycline (512 µg ml^−1^) with amphotericin B enhanced the antifungal activity of the latter against biofilms of *Candida* spp. Similarly, Ku, Palanisamy & Lee (2010) showed that at a concentration of 2,048 µg ml^−1^, tigecycline caused a >50% reduction in the growth of planktonic cells and 84.2% reduction in the metabolic activity of mature *C. albicans* biofilms. Furthermore, the effects of combinations of tigecycline and antifungal agents depended on the type and concentration of the latter.

Mechanistically, tetracyclines are thought to act synergistically with antifungal drugs by enhancing penetration of the latter through biofilms and inducing intracellular calcium release, rather than directly effecting antifungal uptake and efflux (Shi et al., 2010). Furthermore, the effects of tetracyclines have also been associated with the lack of a diauxic shift, which is related to a loss of mitochondrial function due to the similarity between the bacterial and mitochondrial ribosomes. A lack of functional mitochondria would affect sterol metabolism and reduce ergosterol levels. Susceptibility to amphotericin B is further enhanced by its direct actions, specifically its interaction with the fungal membrane and inhibition of ergosterol synthesis (Oliver et al., 2008). Interestingly, it was observed at least one efficient interaction when using combinations of echinocandins and tigecycline against mature and developing biofilms of *C. albicans.* This could be explained by the inhibition of ergosterol synthesis, which ultimately increases permeability allowing antifungal entry (Miceli, Bernardo & Lee, 2009). However, the molecular mechanisms by which tetracyclines and antifungals exert antifungal activity against *C. albicans* remain unclear.

Azoles, especially fluconazole, are the most commonly used antifungal agents for the prevention and treatment of systemic and superficial fungal infections, including candidiasis. These agents are effective, have low toxicity, and are cost-beneficial. However, increasing resistance to azoles has recently arisen among both planktonic and biofilm form of *C. albicans* isolates (Sheehan, Hitchcock & Sibley, 1999; Ramage et al., 2012; Taff et al., 2013; Chandra & Mukherjee, 2015). This study and others have reported that *C. albicans* biofilms may be 4000 times more resistant to fluconazole than related planktonic forms, thus presenting a challenge to the successful use of fluconazole monotherapy (Ramage et al., 2001; Ramage et al., 2002). Despite many reports in which tetracyclines acted synergistically with azoles against *C. albicans* (Miceli, Bernardo & Lee, 2009; Fiori & Van Dijck, 2012; Gao et al., 2013; Liu et al., 2014), in this study, combinations of tigecycline and azoles exerted minimal effects against planktonic cells and mature and developing biofilms. Ku, Palanisamy & Lee (2010) also showed that tigecycline appeared to increase the antifungal effects of fluconazole, but the reduction in metabolic activity by this combination was not statistically different from tigecycline alone at high concentration (512 µg ml^−1^). Hence, these data suggest that the effects of various combinations might be *Candida*-specific, and combinations of azoles and tigecycline should be avoided.

As demonstrated by previous studies and this study, tigecycline alone does not generally exhibit antifungal activity. However, when used at high concentrations, this agent can increase the antifungal activities of amphotericin B and echinocandins. Nevertheless, this study indicated that combinations of tigecycline with azoles reduced the antifungal activities of the latter against both biofilms and planktonic cells of *C. albicans*. In additon, it should be noted that combined use of antibacterial and antifungal drugs in vivo, could be resulted with increased propagation of fungal species, sometimes leading to gastrointestinal overgrowth or superficial infections (Azevedo et al., 2015).

To date, this is the first report of combination therapies involving itraconazole and anidulafungin with tigecycline. Here, tigecycline did not increase the activity of itraconazole against planktonic or biofilm cells, and in fact decreased antibiofilm activity against mature biofilms. Although combinations of anidulafungin and tigecycline did not affect planktonic cells, tigecycline was found to decrease the MIC values of anidulafungin by up to eightfold against preformed biofilms due to reduced ergosterol levels and increased cell wall damage (Oliver et al., 2008).

## Conclusions

Consequently, these results suggest that high concentrations of tigecycline, an antimicrobial lock solution, in combination with amphotericin B and echinocandins, might effectively combat device-related *C. albicans* biofilm infections. In accordance with the ALT principle, the combination of tigecycline with these antifungals might effectively target both bacterial and fungal biofilm infections. Nevertheless, further investigations are needed to confirm tigecycline as a component of ALT.

##  Supplemental Information

10.7717/peerj.5263/supp-1 Supplemental Information 1 MIC and SMIC results, biofilm formation assays, combination assays

10.7717/peerj.5263/supp-2 Supplemental Information 2 Checkerboard scheme

## Additional Information and Declarations

### Data Availability

The following information was supplied regarding data availability: The raw data are provided in the Supplemental Files.
