# Supplementary material for: In vitro activities of antifungals alone and in combination with tigecycline against Candida albicans biofilms
Source: PeerJ. 2018 Jul 25;6:e5263. doi: 10.7717/peerj.5263 (PMC6064206; doi:10.7717/peerj.5263)
Supplement: Supplemental Information 2 [file peerj-06-5263-s002.docx]

| AMB+TIG | AMB+TIG | AMB+TIG | AMB+TIG | AMB+TIG | AMB+TIG | CAS+TIG | CAS+TIG | CAS+TIG | CAS+TIG | CAS+TIG | CAS+TIG |
| --- | --- | --- | --- | --- | --- | --- | --- | --- | --- | --- | --- |
| AMB | AMB | AMB | AMB | AMB | AMB | CAS | CAS | CAS | CAS | CAS | CAS |
| FLC+TIG | FLC+TIG | FLC+TIG | FLC+TIG | FLC+TIG | FLC+TIG | TIG | TIG | TIG | TIG | TIG | TIG |
| FLC | FLC | FLC | FLC | FLC | FLC | CONTROL | CONTROL | CONTROL | CONTROL | CONTROL | CONTROL |
| ITC+TIG | ITC+TIG | ITC+TIG | ITC+TIG | ITC+TIG | ITC+TIG |  |  |  |  |  |  |
| ITC | ITC | ITC | ITC | ITC | ITC |  |  |  |  |  |  |
| ANI+TIG | ANI+TIG | ANI+TIG | ANI+TIG | ANI+TIG | ANI+TIG |  |  |  |  |  |  |
| ANI | ANI | ANI | ANI | ANI | ANI |  |  |  |  |  |  |

AMB: Amphotericin B, FLC: Fluconazole, ITC: Itraconazole, ANI: Anidulafungin, CAS: Caspofungin, TIG: Tigecycline
